# BIC-Based Silicon Metasurfaces for Chiral Response and Tunable Chiral Absorption

**DOI:** 10.3390/nano16120759

**Published:** 2026-06-17

**Authors:** Hao Huang, Qun Ren

**Affiliations:** 1 School of Microelectronics, Tianjin University, Tianjin 300072, China; huanghao_@tju.edu.cn; 2 School of Electrical and Information Engineering, Tianjin University, Tianjin 300072, China

**Keywords:** metasurface, bound states in the continuum, chiral response, graphene, C points

## Abstract

Strong chiral responses in planar dielectric metasurfaces are important for polarization-selective nanophotonic devices, but achieving large and reversible circular dichroism (CD) in simple dielectric structures remains challenging. This work proposes a symmetry-broken silicon metasurface that realizes near-infrared chiral response based on bound states in the continuum (BICs). The unit cell consists of a silicon nanoblock with two through-air grooves. The in-plane displacement of the air grooves breaks the C_2_ rotational symmetry and splits the BIC-related polarization singularity into two circularly polarized points (C points) with opposite handedness. By further introducing out-of-plane tilting, one of the C points is shifted to the Г point, enabling spin-selective coupling between normally incident circularly polarized light and the quasi-BIC mode. Reversing the out-of-plane tilt switches the sign of CD, with values reaching −0.98 and 0.98, approaching the theoretical limits of ±1. Under oblique incidence, the structure can also exhibit near-limit CD responses. Finally, by introducing graphene, the structure achieves tunable circular-polarization-selective absorption, with the absorption of CD approaching the theoretical limits of ±0.5 for the coupled system. This work provides a new design idea for compact chiral nanophotonic materials by using symmetry breaking to control spin-selective quasi-BIC coupling and tunable chiral absorption.

## 1. Introduction

Optical metasurfaces, composed of subwavelength artificial units arranged at interfaces, provide a compact route to control the amplitude, phase, polarization, and propagation direction of light [1,2,3]. Compared with conventional bulk optical elements, metasurfaces allow the desired optical response to be encoded into planar nanostructures, which is attractive for integrated and miniaturized photonic systems. In particular, all-dielectric metasurfaces based on high-index materials such as silicon have received extensive attention because their electric and magnetic Mie resonances can be engineered with relatively low Ohmic loss in the near-infrared and visible spectral regions [4,5,6,7]. These properties make dielectric metasurfaces a useful platform for polarization control, spectral filtering, light–matter interaction enhancement, and nanoscale optical devices.

Chiral light–matter interaction is another important topic in nanophotonics. Circular dichroism (CD), which describes the different optical responses to left- and right-circularly polarized light, is widely used for polarization-selective optics, chiral sensing, and photonic detection [8]. Natural chiral materials usually exhibit weak optical activity because the interaction length and chiral polarizability are limited at the nanoscale. Artificial chiral metasurfaces can enhance this response by introducing structural handedness or extrinsic chirality, and various planar, multilayer, and three-dimensional chiral nanostructures have been proposed to produce strong optical rotation or CD [9,10,11,12,13,14]. However, many of these designs rely on complex chiral geometries, multiple patterned layers, or strong polarization conversion. For practical nanophotonic devices, it remains desirable to realize strong and controllable chiral responses using simple dielectric meta-atoms with clear physical mechanisms.

Bound states in the continuum (BICs) provide another route for enhancing optical resonances in dielectric nanostructures. A BIC is a localized eigenstate embedded in the radiation continuum but decoupled from outgoing radiation channels [15,16,17]. In periodic photonic structures, symmetry-protected BICs can appear at the Γ point, where the mode cannot radiate into free space because of symmetry mismatch. Once the structural symmetry is perturbed, the ideal BIC is converted into a quasi-BIC with finite radiative loss, leading to a sharp Fano resonance and strong field localization [18,19,20,21,22]. Beyond their high-quality-factor(high-*Q*) spectral response, BICs are also associated with polarization singularities in momentum space. The far-field polarization around a BIC can form a vortex carrying a topological charge, and symmetry perturbation can further split or move the corresponding singularities [23,24,25]. This connection between BICs and momentum-space polarization topology offers a useful physical picture for designing polarization-selective metasurfaces.

Recent studies have shown that chiral quasi-BICs can generate strong CD without relying solely on conventional three-dimensional chiral geometries or efficient cross-polarization conversion [26,27,28]. In such systems, the chiral response can originate from the spin-selective coupling between circularly polarized light and a BIC-derived resonance. When circular polarization, namely, circularly polarized points (C points), is controlled in momentum space, one handedness of circular polarization can couple strongly to the quasi-BIC mode, while the opposite handedness remains weakly coupled. This mechanism is attractive for planar nanophotonic devices because it links the macroscopic chiral response to C points.

The integration of active materials also provides another degree of freedom for chiral metasurfaces. Graphene is a two-dimensional material whose optical conductivity can be adjusted through the Fermi level (*E*_F_), enabling dynamic modulation from the terahertz to the infrared range [29,30,31,32,33,34,35]. When graphene is combined with resonant nanostructures, the enhanced near field can increase light absorption in the atomically thin layer, and critical-coupling-like behavior may be obtained under suitable loss and radiation conditions [36,37]. Electrically tunable graphene-loaded antennas and metasurfaces have further demonstrated that graphene can provide an efficient route for modulating resonance strength, damping, and absorption spectra [38,39]. Therefore, coupling graphene with chiral quasi-BIC metasurfaces may allow the conversion of transmission-type chiral selectivity into tunable circular-polarization-selective absorption.

In this work, we propose a near-infrared silicon metasurface for strong chiral optical response based on symmetry-broken quasi-BICs. The unit cell consists of a silicon nanoblock with two through-air grooves. By changing the relative positions of the grooves, the in-plane C_2_ rotational symmetry is broken, causing the polarization singularity at the Γ point to split into two circularly polarized C points in momentum space. By further introducing an out-of-plane tilt, one of the C points can be shifted to the Γ point, enabling strong spin-selective coupling under normal incidence. As a result, near-unity CD with opposite signs can be obtained by reversing the tilt direction. Multipole decomposition and near-field distributions reveal that the chiral quasi-BIC response is mainly governed by a magnetic-dipole-dominated resonance. The angular response is also investigated to clarify the relation between the C point distribution and the incidence direction. Finally, a monolayer graphene sheet is introduced on the metasurface to realize tunable circular-polarization-selective absorption. The proposed structure provides a silicon-based platform for chiral filtering, polarization-sensitive absorption, and tunable nanophotonic devices.

## 2. Materials and Methods

**Figure 1:**
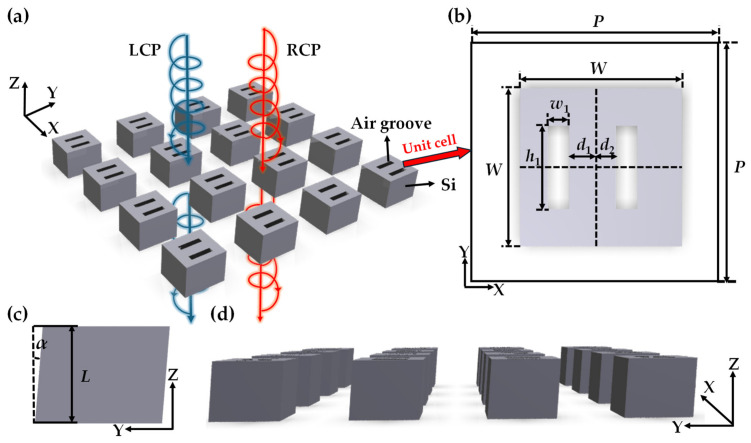
Schematic of the proposed silicon metasurface. (**a**) Three-dimensional view of the periodic metasurface under left-circularly polarized(LCP) and right-circularly polarized(RCP) illumination. (**b**) Top view of the unit cell with two air grooves, where the dashed lines denote the central reference lines. (**c**) Cross-sectional view of the tilted silicon unit in the *y*-*z* plane, where the dashed line indicates the original vertical reference line before tilting, and *α* denotes the tilting angle. (**d**) Side view of the metasurface array.

The metasurface investigated in this study is composed of silicon with a refractive index of 3.48, and its overall structural schematic is shown in Figure 1. The metasurface consists of periodically arranged silicon cuboid unit cells, as illustrated in Figure 1a. Figure 1b presents the top view of the unit cell, where the lattice period is *P* = 750 nm and the in-plane dimension of the silicon unit is *W* = 380 nm. The thickness of the silicon unit is *L* = 290 nm, as shown in Figure 1c. To modulate the in-plane symmetry of the structure, two air grooves penetrating through the silicon unit along the thickness direction are introduced into each unit cell. The length and width of each groove are *h*_1_ = 200 nm and *w*_1_ = 50 nm, respectively. The two air grooves are displaced from the center of the unit cell along the *x*-direction by *d*_1_ = 75 nm and *d*_2_ = 35 nm, respectively, thereby introducing an in-plane asymmetric perturbation.

On the basis of the broken in-plane C_2_ rotational symmetry, the silicon unit is further tilted out of plane to reduce the structural symmetry along the *z*-direction. Figure 1c shows the cross-sectional schematic of the unit cell in the *y*-*z* plane, where the out-of-plane tilting angle is *α* = 0.08 rad. Figure 1d presents the corresponding side-view schematic of the metasurface array. In this study, the chiral responses of the structure to normally incident left-circularly polarized (LCP) and right-circularly polarized (RCP) light propagating along the -*z* direction are mainly investigated in the wavelength range of 1375–1380 nm.

All numerical simulations were performed using the finite element method in COMSOL Multiphysics 6.3. A single unit cell of the silicon metasurface was modeled, and periodic boundary conditions were applied along the *x*- and *y*-directions to represent an infinite periodic array. Perfectly matched layers were placed along the propagation direction to suppress nonphysical reflections. For oblique incidence, the in-plane wave vector was defined as *k*_x_ = *k*_0_sin*θ*cos*ϕ* and *k*_y_ = *k*_0_sin*θ*sin*ϕ*, where *θ* and *ϕ* represent the incident elevation angle and incident azimuth angle.

## 3. Results and Discussion

### 3.1. Eigenmode Characteristics of the Symmetry-Protected BIC

**Figure 2:**
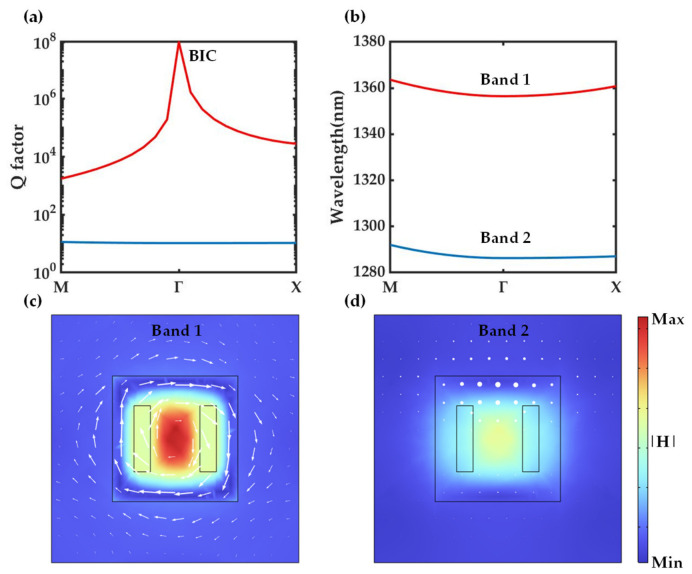
Eigenmode analysis of the symmetry-preserved metasurface. (**a**) Quality factors (*Q* factors) and (**b**) eigenmode wavelengths of Band 1 and Band 2 along the M-Γ-X high-symmetry path, where the red and blue lines in (**a**) represent the *Q* factors of the bound states in the continuum(BIC) mode and the non-BIC mode, respectively. (**c**,**d**) Electric-field vector distributions and magnetic-field amplitude |H| distributions of (**c**) Band 1 and (**d**) Band 2 at the Γ point, with the white arrows in (**c**) indicating the in-plane electric-field directions and the white dots in (**d**) indicating electric-field vectors mainly oriented along the *z* direction.

When the metasurface unit cell preserves complete in-plane C2 rotational symmetry and out-of-plane mirror symmetry, namely when the structural parameters satisfy *d_1_* = *d_2_* = 75 nm and the out-of-plane tilting angle is α = 0 rad, the structure can be regarded as a reference configuration for a symmetry-protected BIC. Based on this configuration, finite-element electromagnetic simulations were performed to calculate the quality factors of the eigenmodes along the M-Г-X high-symmetry path, as shown in Figure 2a.

As shown in Figure 2a, the eigenmode corresponding to the red curve exhibits a rapid increase in quality factor as the wave vector approaches the Г point from the M point, and a sharp peak appears near the Г point. When the wave vector further moves from the Г point toward the X point, the quality factor decreases again and returns to a finite value. This behavior indicates that, at the Г point, the coupling between this mode and the free-space radiation channels is suppressed by the structural symmetry, resulting in strongly reduced radiative loss and a theoretically divergent quality factor. This is a typical feature of a symmetry-protected BIC.

In contrast, the eigenmode corresponding to the blue curve in Figure 2a maintains a low and finite quality factor over the entire M-Г-X path, indicating that it can effectively couple to external radiation channels and can therefore be identified as a conventional radiative mode. The two eigenmodes also exhibit distinct resonant wavelengths. The red band is located near 1360 nm and is denoted as Band 1, whereas the blue band is located near 1290 nm and is denoted as Band 2, as shown in Figure 2b.

To further analyze the field distributions of the two modes, Figure 2c,d show the electric-field vector distributions and magnetic-field amplitude |H| distributions of Band 1 and Band 2 at the Г point, respectively. For Band 1, the electric-field vectors exhibit a distinct circular distribution around the silicon unit, while the mag-netic field is mainly localized at the center of the structure, indicating strong local field confinement. For Band 2, the electric-field distribution does not show a similar circular topological feature, and the magnetic field is relatively more dispersed, with a significantly weaker local enhancement than that of Band 1. These results further indicate that Band 1 is closely associated with the formation of the symmetry-protected BIC, whereas Band 2 is more similar to an ordinary radiative mode.

### 3.2. Chiral Response Induced by Symmetry Breaking

**Figure 3:**
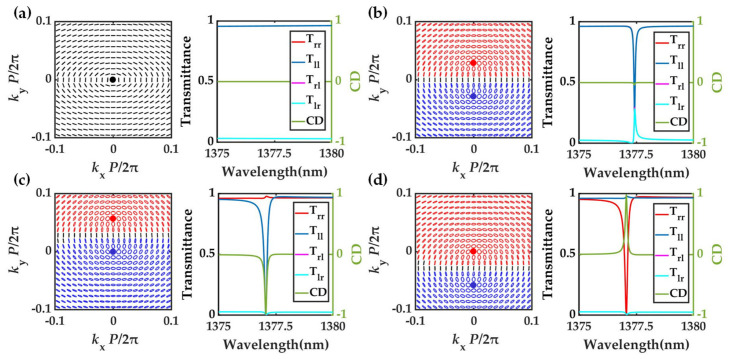
Far-field polarization distributions in momentum space and circular-polarization transmission spectra under different symmetry perturbations. (**a**) *d*_1_ = *d*_2_ = 75 nm, *α* = 0 rad. (**b**) *d*_1_ = 75 nm, *d*_2_ = 35 nm, *α* = 0 rad. (**c**) *d*_1_ = 75 nm, *d*_2_ = 35 nm, *α* = −0.08 rad. (**d**) *d*_1_ = 75 nm, *d*_2_ = 35 nm, *α* = 0.08 rad. The right panels show T_rr_, T_ll_, T_rl_, T_lr_ and circular dichroism(CD) spectra. In the left panels of (**a**–**d**), the red and blue polarization ellipses and circular dots represent opposite elliptical polarization states and the corresponding circular polarization points (C points), respectively, while the black lines and black dot denote linear polarization and a polarization singularity, respectively.

When the structural parameters satisfy *d*_1_ = *d*_2_ = 75 nm and α = 0 rad, the metasurface preserves both the in-plane C_2_ rotational symmetry and the out-of-plane mirror symmetry. Under this highly symmetric configuration, Band 1 supports an ideal symmetry-protected BIC at the Г point. To reveal the far-field polarization characteristics of this mode, the polarization-state distribution in momentum space near the Г point was calculated, as shown in the left panel of Figure 3a. In this symmetric configuration, the far-field polarization states mainly exhibit linear polarization, with a polarization singularity located at the Г point. At this singular point, the radiation channel is forbidden by symmetry, and therefore the far-field polarization direction is undefined. The surrounding linear polarization directions form a vortex-like distribution around the singularity, indicating that the BIC mode carries a well-defined topological charge in momentum space.

The right panel of Figure 3a shows the circular-polarization transmission spectra of this configuration under normal incidence. Here, T_rr_ and T_ll_ denote the co-polarized transmission components for RCP and LCP waves, respectively, while T_rl_ and T_lr_ represent the cross-polarized conversion components. T_rl_ represents the transmission coefficient from incident LCP light to transmitted RCP light, whereas T_lr_ represents the transmission coefficient from incident RCP light to transmitted LCP light. It can be observed that both T_rr_ and T_ll_ are close to 1, whereas T_rl_ and T_lr_ are nearly zero. According to the definition of CD, CD can be expressed as follows [40]:

(1) $$\mathrm{CD}=\frac{{\left|T_{\mathrm{rl}}\right|}^{2}+{\left|T_{\mathrm{ll}}\right|}^{2}-{\left|T_{\mathrm{lr}}\right|}^{2}-{\left|T_{\mathrm{rr}}\right|}^{2}}{{\left|T_{\mathrm{rl}}\right|}^{2}+{\left|T_{\mathrm{ll}}\right|}^{2}+{\left|T_{\mathrm{lr}}\right|}^{2}+{\left|T_{\mathrm{rr}}\right|}^{2}}$$

According to this definition, the CD value of this structure is close to zero, indicating that the fully symmetric structure does not exhibit a distinct chiral response.

When the structural parameters are changed to *d*_1_ = 75 nm and *d*_2_ = 35 nm, the in-plane C_2_ rotational symmetry is broken, while the out-of-plane mirror symmetry is still preserved. In this case, the original polarization singularity located at the Г point splits into two circular polarization points, namely C points, which are symmetrically distributed with respect to the Г point, as shown in the left panel of Figure 3b. These two C points are located near *k*_x_*P*/2π = 0 and *k*_y_*P*/2π = ±0.03, corresponding to circular polarization states with opposite handedness. This result indicates that the breaking of in-plane symmetry can transform the BIC into a quasi-BIC and induce localized circular polarization states in momentum space. However, because the out-of-plane mirror symmetry remains unbroken, the structure still does not exhibit an obvious overall chiral response under normal incidence. As shown in the right panel of Figure 3b, T_rr_ and T_ll_ remain nearly identical, and the CD value is still close to zero.

After further introducing an out-of-plane tilt angle of *α* = −0.08 rad, the out-of-plane mirror symmetry is broken, and the two C points shift simultaneously in momentum space, as shown in the left panel of Figure 3c. In this case, the C-point moves close to the Г point, making the far-field polarization state under normal incidence approach LCP. Accordingly, as shown in the right panel of Figure 3c, T_ll_ decreases sharply at the resonant wavelength and approaches zero, whereas T_rr_ remains at a high transmission level. Meanwhile, the cross-polarized components T_rl_ and T_lr_ remain close to zero. The calculated CD value is approximately −0.98, which is close to the theoretical limit of −1, demonstrating a strong LCP-selective response under this tilting condition.

When the out-of-plane tilt angle is reversed to *α* = 0.08 rad, the two C points shift in the opposite direction, as shown in the left panel of Figure 3d. In this case, the C_+_ point moves close to the Г point, making the far-field polarization state under normal incidence approach RCP. Correspondingly, T_rr_ is significantly suppressed at resonance, while T_ll_ remains close to 1, as shown in the right panel of Figure 3d. As a result, a CD value of approximately +0.98 is obtained. These results indicate that the sign of the chiral response can be reversed by controlling the direction of the out-of-plane tilt. The near-unity circular dichroism originates from the overlap between the C point and the Г point, together with the selective resonant coupling between the quasi-BIC mode and a specific circular polarization state.

**Figure 4:**
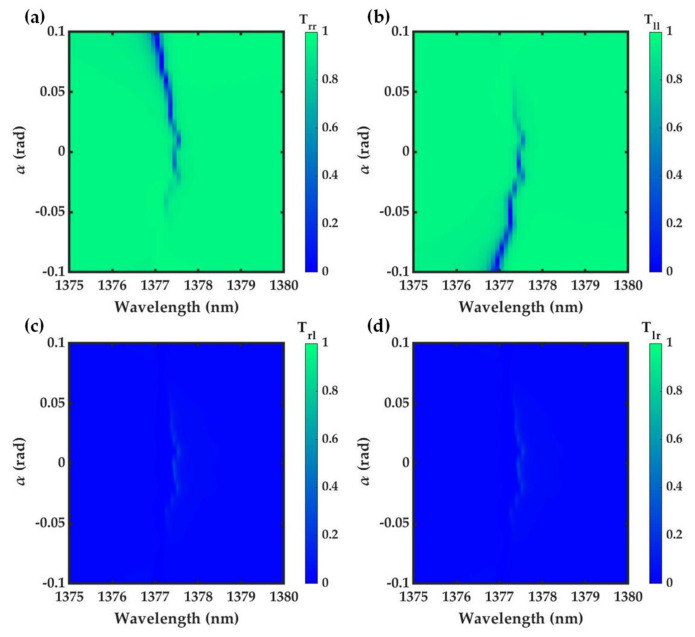
Evolution of the circular-polarization transmission components as functions of wavelength and out-of-plane tilt angle *α* for the metasurface with *d*_1_ = 75 nm and *d*_2_ = 35 nm: (**a**) T_rr_, (**b**) T_ll_, (**c**) T_rl_, (**d**) T_lr_.

Under fixed in-plane asymmetric parameters of *d*_1_ = 75 nm and *d*_2_ = 35 nm, the influence of the out-of-plane tilt angle α on the circular-polarization transmission response was further investigated. Figure 4a–d show the two-dimensional distributions of the co-polarized transmission components T_rr_ and T_ll_, as well as the cross-polarized transmission components T_rl_ and T_lr_, as functions of wavelength and *α* in the wavelength range of 1375–1380 nm. The wavelength was swept with a step size of 0.01 nm, and the color maps were plotted directly from the raw simulated data without additional smoothing or interpolation.

As shown in Figure 4a,b, T_rr_ and T_ll_ exhibit pronounced mirror-symmetric response characteristics with respect to *α*. When *α* > 0, the co-polarized transmission component T_rr_ for RCP incidence shows a distinct transmission dip near 1377 nm. As *α* increases, the linewidth of this transmission dip gradually broadens, indicating enhanced radiative coupling between this polarization channel and the quasi-BIC mode. In contrast, the co-polarized transmission component T_ll_ for LCP incidence remains at a high transmission level without obvious suppression. When *α* < 0, the response behavior is reversed: T_ll_ exhibits a pronounced transmission dip around the same wavelength, whereas T_rr_ maintains a high transmittance. This interchange of the co-polarized transmission response induced by reversing the tilting direction demonstrates that the out-of-plane asymmetry can effectively control the selective coupling of the structure to LCP and RCP waves.

It should be noted that the slight non-monotonic features in the resonance trajectory are present in the raw simulated data. These features may be associated with the α-dependent evolution of the quasi-BIC resonance, including variations in the resonance position, linewidth, and coupling strength to the incident circular polarization.

By comparison, as shown in Figure 4c,d, the cross-polarized transmission components T_rl_ and T_lr_ remain at low levels over the entire parameter range, with only slight variations near the resonance. This indicates that the chiral response of the structure mainly originates from the selective transmission difference between the two co-polarized circular-polarization channels, rather than from significant cross-polarization conversion. As |*α*| increases, the transmission contrast between the co-polarized channels is further enhanced, leading to a circular dichroism response that gradually approaches the ideal limit.

### 3.3. Multipolar Origin of the Chiral Quasi-BIC

**Figure 5:**
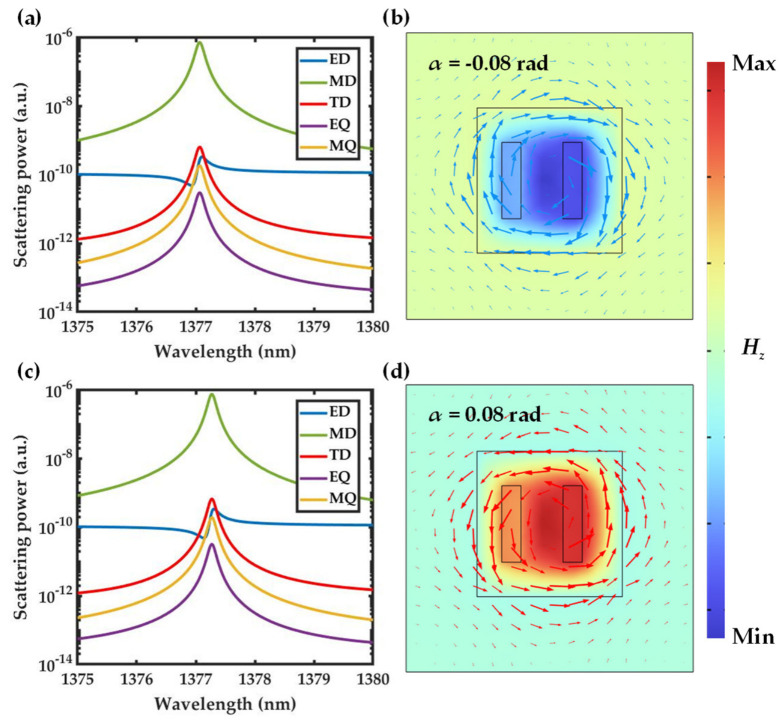
Multipolar decomposition and near-field distributions of the chiral quasi-BIC modes. (**a**,**b**) Results for *α* = −0.08 rad under LCP incidence. (**c**,**d**) Results for *α* = 0.08 rad under RCP incidence. The structural parameters are *d*_1_ = 75 nm and *d*_2_ = 35 nm. The arrows indicate the in-plane electric-field vectors, and the color maps represent *H_z_*.

To further analyze the physical origin of the chiral quasi-BIC mode, multipole decomposition and near-field distribution analysis were performed for two configurations with opposite out-of-plane tilting directions. Figure 5a,b show the multipole scattering powers and electromagnetic field distribution. Five scattering contributions are taken into account, namely the electric dipole (ED), magnetic dipole (MD), toroidal dipole (TD), electric quadrupole (EQ), and magnetic quadrupole (MQ). The scattering power of each multipole can be calculated as follows [40]:

(2) $$P=\frac{1}{i\omega }{∭}_{V}jd^{3}r,$$

(3) $$M=\frac{1}{2c}{∭}_{V}\left(r\times j\right)d^{3}r,$$

(4) $$T=\frac{1}{10c}{∭}_{V}\left[(r\cdot j)r-2r^{2}j\right]d^{3}r,$$

(5) $$EQ_{\alpha ,\beta }=\frac{1}{10c}{∭}_{V}\left[(r_{\alpha }j_{\beta }+r_{\beta }j_{\alpha })-\frac{2}{3}(r\cdot j)\right]d^{3}r,$$

(6) $$MQ_{\alpha ,\beta }=\frac{1}{3c}{∭}_{V}\left[{\left(r\times j\right)}_{\alpha }r_{\beta }+{\left(r\times j\right)}_{\beta }r_{\alpha }\right]d^{3}r,$$

where *ω* represents angular frequency, *c* is the speed of light, *α*,*β* = *x*,*y*,*z*, ***j*** are surface current density.

For the structure with *d*_1_ = 75 nm, *d*_2_ = 35 nm, and *α* = −0.08 rad, the multipole decomposition results are shown in Figure 5a. At the resonant wavelength of approximately 1377.1 nm, the MD contribution is dominant and is significantly stronger than the ED, TD, EQ, and MQ contributions. This indicates that the quasi-BIC mode is mainly governed by the MD response. Figure 5b further presents the corresponding in-plane electric-field vectors and *H*_z_ distribution. The electric-field vectors form a pronounced closed circulating pattern around the center of the silicon unit, while *H*_z_ is strongly localized in the central region of the structure. These features are consistent with the typical field distribution of a magnetic dipole mode.

Figure 5c,d show the corresponding results for the structure with *d*_1_ = 75 nm, *d*_2_ = 35 nm and *α* = 0.08 rad. Similar to the case of *α* = −0.08 rad, this configuration also exhibits a multipole scattering response dominated by the MD component near 1377.1 nm, as shown in Figure 5c. However, owing to the reversal of the out-of-plane tilting direction, the in-plane electric-field circulation in Figure 5d is opposite to that in Figure 5b, leading to a localized *H*_z_ enhancement with the opposite sign. These results indicate that the chiral quasi-BIC modes in the two oppositely tilted configurations are both mainly derived from the MD response, while the reversal of the out-of-plane tilting direction changes the electric-field circulation and the sign of *H*_z_, thereby producing opposite circular-polarization-selective responses.

### 3.4. Angular Dependence of the Chiral Response

**Figure 6:**
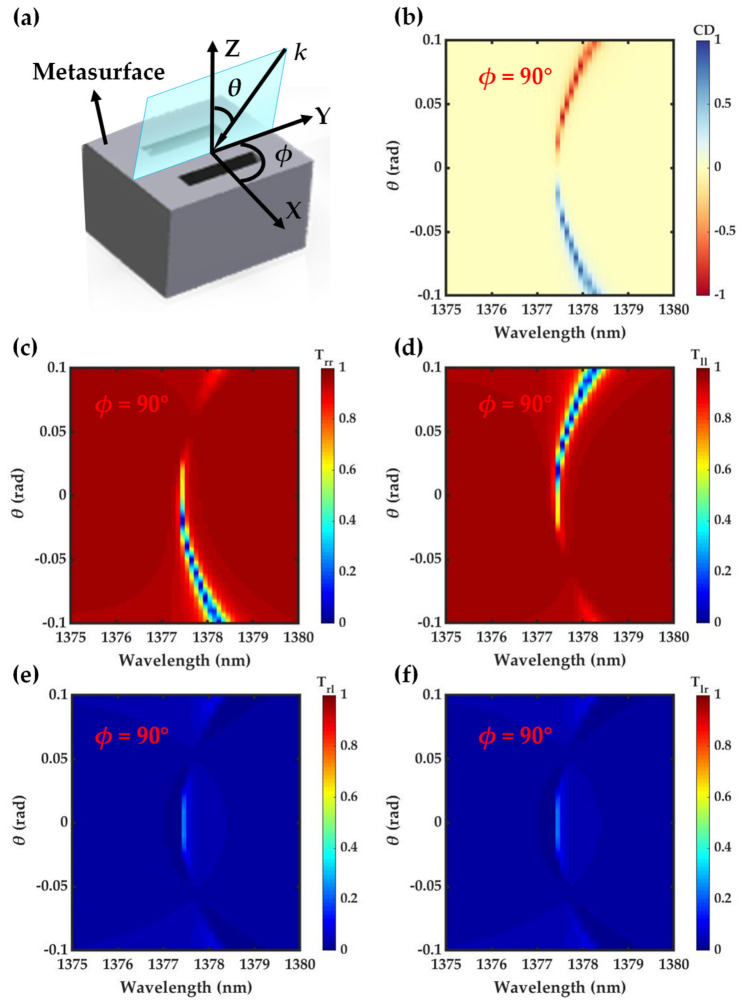
Oblique-incidence chiral response of the in-plane symmetry-broken metasurface with *d*_1_ = 75 nm, *d*_2_ = 35 nm and *α* = 0 rad. (**a**) Schematic of oblique incidence, where *θ* and *ϕ* denote the incident polar angle and azimuthal angle, respectively. (**b**) CD spectra as a function of wavelength and *θ* at *ϕ* = 90°. (**c**,**d**) Co-polarized transmission components T_rr_ and T_ll_. (**e**,**f**) Cross-polarized transmission components T_rl_ and T_lr_.

For the configuration in which only the in-plane C_2_ rotational symmetry is broken while the out-of-plane mirror symmetry is preserved, namely *d*_1_ = 75 nm, *d*_2_ = 35 nm and *α* = 0 rad, the metasurface shows a weak circular dichroism response under normal incidence. However, when the incident direction deviates from the surface normal, the incident wave vector can match the split circular-polarization C points in momentum space, thereby inducing a pronounced angle-dependent chiral response. Figure 6a defines the incident-angle parameters under oblique incidence, where *θ* denotes the polar angle of the incident wave vector with respect to the surface normal, and *ϕ* denotes the azimuthal angle of its projection in the *x*-*y* plane.

According to the C-point distribution discussed above, when only the in-plane C_2_ rotational symmetry is broken, the two circular-polarization C points are located along the *k*_x_*P*/2π = 0 axis and are symmetrically distributed along the *k*_y_ direction. Therefore, *ϕ* = 90° is selected so that the incident wave vector deviates from the Г point along the *k*_y_ direction in momentum space. This allows the circular-polarization-selective response associated with the C points to be examined.

Figure 6b shows the variation of CD as a function of wavelength and incident angle *θ*. As |*θ*| increases, the incident wave vector gradually approaches the positions of the two C points, leading to enhanced circular-polarization-selective coupling. As a result, CD values close to ±1 are obtained near *θ* ≈ ±0.06 rad. When |*θ*| further increases and the incident wave vector moves away from the C-point positions, |CD| decreases, while the corresponding resonance bandwidth becomes broader. These results indicate that the chiral response of the structure is determined not only by structural symmetry breaking, but also by the position of the incident wave vector in momentum space.

To further clarify the mechanism responsible for the strong CD response, Figure 6c–f present the wavelength- and angle-dependent variations of T_rr_, T_ll_, T_rl_ and T_lr_, respectively. As shown in Figure 6c,d, T_rr_ and T_ll_ exhibit clear complementary behavior. For one oblique incident direction, T_rr_ shows a pronounced transmission dip, while T_ll_ remains at a high transmission level. For the opposite incident direction, T_ll_ exhibits a strong transmission dip, whereas T_rr_ remains highly transmissive. This indicates that the two opposite oblique incident directions selectively excite circularly polarized quasi-BIC modes with opposite handedness, resulting in a significant transmission contrast between RCP and LCP waves.

In contrast, the cross-polarized transmission components T_rl_ and T_lr_ in Figure 6e,f remain at low levels over the entire angular scanning range. This suggests that the chiral response under oblique incidence mainly originates from selective coupling in the co-polarized channels, rather than from cross-polarization conversion between the two circular polarization states. These results demonstrate that the strength and sign of the chiral response in the in-plane symmetry-broken metasurface can be effectively controlled by tuning the incident angle *θ*.

### 3.5. Graphene-Assisted Tunable Chiral Absorption

**Figure 7:**
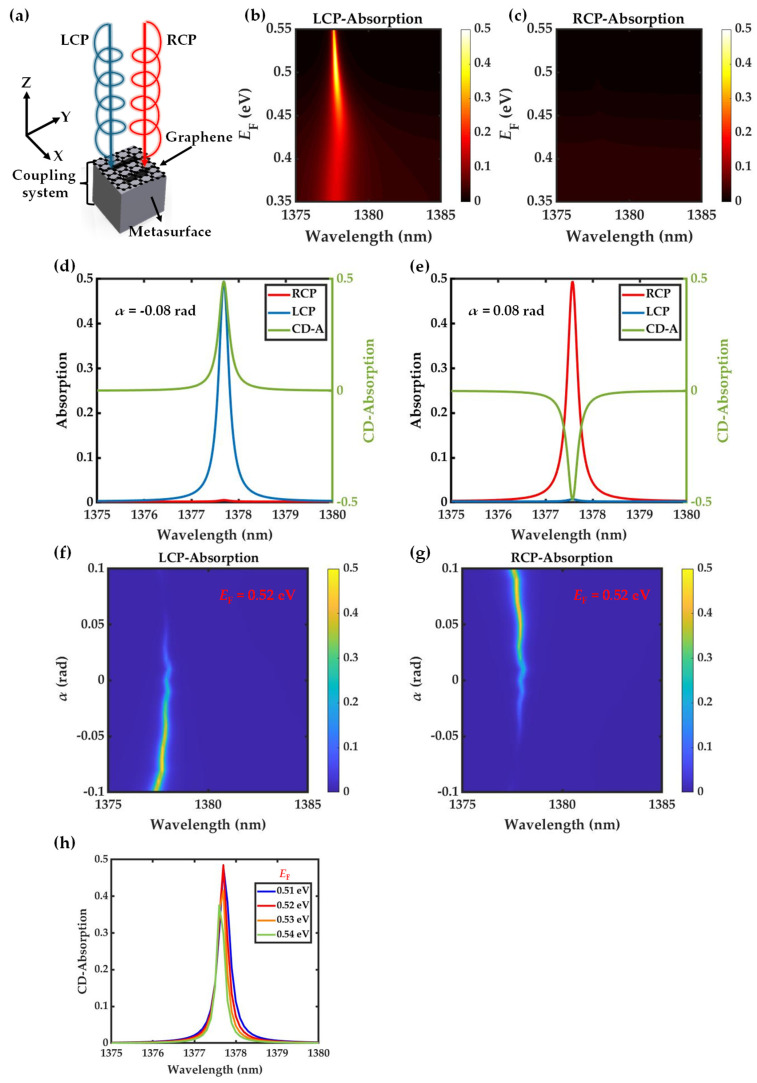
Tunable chiral absorption in the graphene-integrated metasurface. (**a**) Schematic of the graphene–metasurface coupling system under LCP and RCP incidence. (**b**,**c**) Absorption maps for LCP and RCP incidence as functions of wavelength and graphene Fermi level(*E*_F_) for *α* = −0.08 rad. (**d**,**e**) Absorption spectra of LCP and RCP waves and the circular dichroism absorption(CD-A) at *E*_F_ = 0.52 eV for *α* = −0.08 rad and *α* = 0.08 rad, respectively. (**f**,**g**) Absorption maps for LCP and RCP incidence as functions of wavelength and the out-of-plane asymmetry parameter *α* at *E*_F_ = 0.52 eV. The structural parameters are fixed as *d*_1_ = 75 nm and *d*_2_ = 35 nm. (**h**) CD-A spectra for different graphene *E*_F_ of 0.51, 0.52, 0.53, and 0.54 eV.

To further extend the functionality of the metasurface, graphene was introduced into the system. As shown in Figure 7a, a monolayer graphene sheet was placed on top of the silicon metasurface to construct a graphene–metasurface hybrid system. The optical response of graphene strongly depends on its *E*_F_, because tuning *E*_F_ changes the surface conductivity of graphene and consequently modifies the dissipative loss introduced by the graphene layer. The surface conductivity of graphene can be expressed as follows:

(7) $$\begin{array}{l} \sigma =\frac{e^{2}}{4\hbar }\left[\frac{1}{2}+\frac{1}{\pi }\tan^{-1}\left(\frac{\hbar \omega -2E_{F}}{2k_{B}T}\right)-\frac{i}{2\pi }\ln\frac{{\left(\hbar \omega -2E_{F}\right)}^{2}}{{\left(\hbar \omega -2E_{F}\right)}^{2}-4{\left(k_{B}T\right)}^{2}}\right]\\ \;\;+\frac{i}{\omega +i\tau^{-1}}\cdot \frac{2e^{2}k_{B}T}{\pi \hbar^{2}}\ln\left[2\cosh\left(\frac{E_{F}}{2k_{B}T}\right)\right] \end{array},$$

where *k*_B_, *e*, *ħ*, *T*, and *ω* denote the Boltzmann constant, electron charge, reduced Planck constant, room temperature, and angular frequency of the incident light, respectively. *τ* is the carrier relaxation time, which depends on the carrier mobility *μ*, *E*_F_, and Fermi velocity *v*_F_, and can be expressed as follows:

(8) $$\tau =\frac{\mu E_{F}}{ev_{F^{2}}},$$

where *v*_F_ = 10^6^ m/s and *μ* = 10^4^ cm^2^/(V·s). Varying the chemical potential changes the conductivity of graphene, thereby modifying the equivalent dissipative loss rate introduced by the graphene layer.

To clarify the absorption mechanism of the graphene-integrated chiral metasurface, temporal coupled-mode theory was used to describe the resonant coupling process. For a symmetric two-port resonant system under one-sided illumination, the temporal evolution of the resonant amplitude *a* can be written as follows [41]:

(9) $$\frac{da}{dt}=(jw_{0}-\delta -\gamma )a+\sqrt{\delta }S_{\mathrm{in}}e^{j\theta },$$

where *ω*_0_ is the resonant angular frequency, *δ* is the radiative decay rate, *γ* is the dissipative loss rate, *S*_in_ is the incident wave amplitude, and *θ* is the coupling phase. Under steady-state excitation, the resonant amplitude is given by

(10) $$a=\frac{\sqrt{\delta }S_{\mathrm{in}}e^{j\theta }}{j(\omega -\omega_{0})+\delta +\gamma }.$$

The absorption at angular frequency *ω* can therefore be expressed as follows:

(11) $$A=\frac{2\delta \gamma }{{\left(\delta +\gamma \right)}^{2}+{\left(\omega -w_{0}\right)}^{2}}.$$

At resonance, namely *ω* = *ω*_0_, the absorption can be expressed by

(12) $$A=\frac{2\delta \gamma }{{\left(\delta +\gamma \right)}^{2}}$$

Therefore, when the graphene-induced dissipative loss rate *γ* matches the radiative loss rate *δ*, the system satisfies the critical coupling condition, and the absorption reaches the theoretical limit of 0.5.

When the structural parameters are *d*_1_ = 75 nm and *d*_2_ = 35 nm, with an out-of-plane asymmetry parameter of *α* = −0.08 rad, Figure 7b,c show the absorption responses under LCP and RCP incidence as a function of *E*_F_, respectively. It can be observed that, within the wavelength range of 1375–1385 nm, the hybrid system exhibits a pronounced tunable absorption response for LCP light, whereas the absorption for RCP light remains nearly zero. As *E*_F_ increases, the resonant absorption for LCP light first increases and then decreases. Consequently, circular dichroism absorption(CD-A) reaches its maximum near *E*_F_ = 0.52 eV. This indicates that *E*_F_ = 0.52 eV provides the closest loss-matching condition between the graphene-induced dissipative loss and the radiative loss of the chiral quasi-BIC resonance, thereby leading to the maximum chiral absorption. Here, CD-A is defined as CD-A = A_LCP_ − A_RCP_, where A_LCP_ and A_RCP_ denote the absorptance under LCP and RCP incidence, respectively. Figure 7h further shows the CD-A spectra for *E*_F_ = 0.51, 0.52, 0.53, and 0.54 eV. Furthermore, the absorption spectra were calculated for *α* = −0.08 rad and *α* = 0.08 rad, as shown in Figure 7d,e, respectively. For *α* = −0.08 rad, the system exhibits strong absorption for LCP light at approximately 1377.5 nm, whereas the absorption of RCP light is nearly negligible. When *α* = 0.08 rad, the absorption selectivity is reversed: RCP light shows pronounced absorption in the same spectral range, while LCP absorption is suppressed to nearly zero. The corresponding absorption of circular dichroism, denoted as CD-A, approaches opposite extrema in the two cases, demonstrating that the handedness of chiral absorption can be switched by reversing the out-of-plane tilting direction.

In addition, with the *E*_F_ = 0.52 eV, the dependence of the absorption response on the out-of-plane asymmetry parameter *α* was further investigated. As shown in Figure 7f,g, the absorption distributions for LCP and RCP light exhibit an approximately mirror-symmetric relationship with respect to *α* = 0 rad. Specifically, when one circular polarization reaches strong absorption, the absorption of the opposite circular polarization is significantly suppressed. These results indicate that the out-of-plane asymmetry parameter not only determines the sign of the chiral response, but also provides an effective degree of freedom for realizing selective absorption of a target circular polarization. Therefore, by jointly tuning the graphene *E*_F_ and the out-of-plane asymmetry parameter *α*, the proposed structure enables controllable modulation of both the circular-polarization-selective absorption intensity and the handedness of chiral absorption without changing the geometrical dimensions, providing a feasible strategy for tunable chiral absorbers and polarization-sensitive optoelectronic detection systems. In addition, it should be noted that, in the proposed structure, changing *α* modulates the degree of out-of-plane symmetry breaking and shifts the distribution of the C points in momentum space. Under a fixed incidence condition, the matching relationship between the incident wave vector and the C point changes with *α*. Therefore, the resonance wavelength and radiative leakage rate of the chiral quasi-BIC mode, as well as its coupling strength to LCP or RCP incidence, are modified simultaneously. Since these factors do not necessarily vary monotonically with *α*, the extracted resonance positions in the figures may exhibit slight oscillatory shifts.

**Figure 8:**
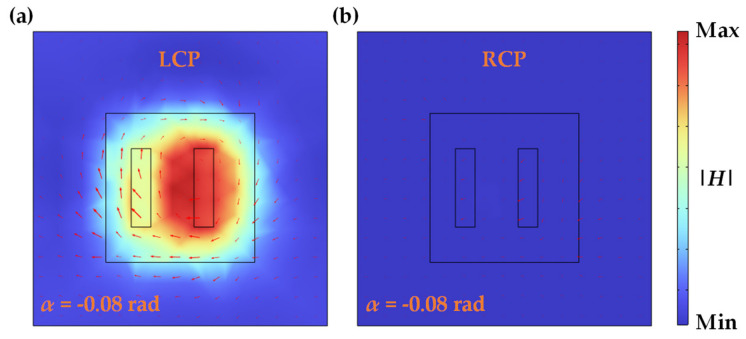
Near-field distributions on the graphene plane under (**a**) LCP and (**b**) RCP incidence at *E*_F_ = 0.52 eV and *α* = −0.08 rad. The color map represents the magnetic-field amplitude |*H*|, and the arrows indicate the in-plane electric field vector.

Furthermore, to further clarify the physical origin of the graphene-induced chiral absorption, the near-field distributions on the graphene plane were analyzed to reveal the overlap between the chiral quasi-BIC mode supported by the silicon nanoblocks and the graphene monolayer. Specifically, as shown in Figure 8a,b, at *E*_F_ = 0.52 eV and a wavelength of 1377.7 nm, we compared the electromagnetic field distributions on the graphene plane under LCP and RCP incidence. The results show that LCP incidence produces much stronger localized electric and magnetic fields on the graphene layer, indicating that the chiral quasi-BIC mode can be efficiently excited by LCP light. In contrast, RCP incidence exhibits much weaker field localization on the graphene plane, suggesting a weaker coupling to the chiral quasi-BIC mode; thus, the graphene-induced dissipation is negligible. The physical origin of this difference lies in the handedness-dependent coupling of the chiral quasi-BIC resonance. Owing to the simultaneous breaking of in-plane and out-of-plane symmetries, the silicon metasurface supports a chiral quasi-BIC mode with a specific circular polarization handedness. This pronounced difference in localized fields leads to a strong circular-polarization-dependent dissipative loss in the graphene layer, thereby giving rise to the selective absorption difference between LCP and RCP light.

To evaluate the fabrication robustness of the proposed chiral quasi-BIC metasurface, a fabrication-tolerance analysis was performed by considering three typical nanofabrication imperfections: rounded groove corners, sidewall-angle variation, and over-etching. The corresponding CD and *Q* factor were calculated to quantify the influence of these geometrical deviations on the chiral response and *Q* factor.

**Figure 9:**
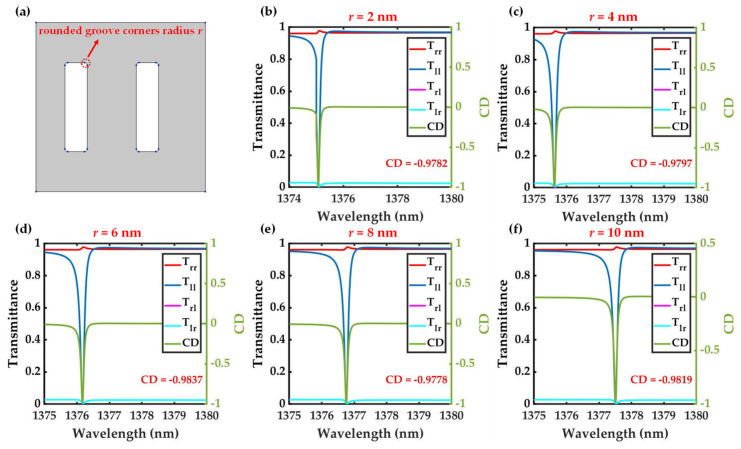
Fabrication-tolerance analysis of the proposed chiral metasurface with rounded groove corners. (**a**) Schematic of the rounded groove corner with radius *r*. (**b**–**f**) Transmission spectra and CD responses for different rounded-corner radius *r* = 2, 4, 6, 8, and 10 nm, respectively.

**Figure 10:**
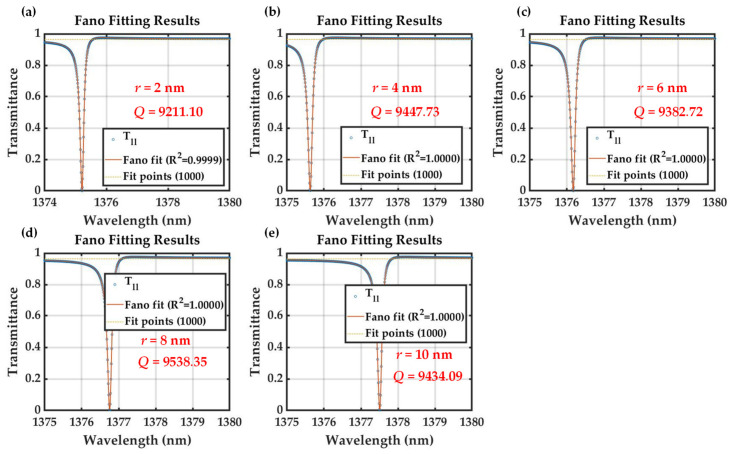
Fano fitting results of the T_ll_ spectra under different rounded-corner radii for the proposed chiral metasurface. (**a**–**e**) Fano fitting results for *r* = 2, 4, 6, 8, and 10 nm, respectively, under *d*_1_ = 75 nm, *d*_2_ = 35 nm, and *α* = −0.08 rad. The extracted *Q* factors are 9211.10, 9447.73, 9382.72, 9538.35, and 9434.09, respectively.

First, we investigated the influence of rounded groove corners on the CD and *Q* factor by taking the chiral metasurface with *d*_1_ = 75 nm, *d*_2_ = 35 nm, and *α* = −0.08 rad as an example. The radius of the rounded groove corner is denoted as *r*, as shown in Figure 9a. We calculated the CD and *Q* factor for *r* = 2, 4, 6, 8, and 10 nm. As shown in Figure 9b–f and Figure 10a–e, where R^2^ represents the fitting accuracy, the corresponding CD values are −0.9782, −0.9797, −0.9837, −0.9778, and −0.9819, respectively; the corresponding *Q* factors are 9211.10, 9447.73, 9382.72, 9538.35, and 9434.09, respectively.

**Figure 11:**
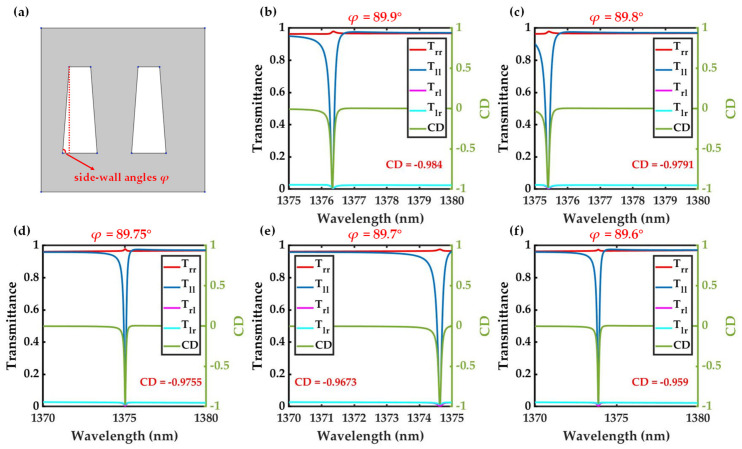
Influence of sidewall-angle variation on the chiral response of the proposed metasurface. (**a**) Schematic of the sidewall angle *φ*. (**b**–**f**) Transmission spectra and CD responses for *φ* = 89.9°, 89.8°, 89.75°, 89.7°, and 89.6°, respectively, under *d*_1_ = 75 nm, *d*_2_ = 35 nm, and *α* = −0.08 rad.

**Figure 12:**
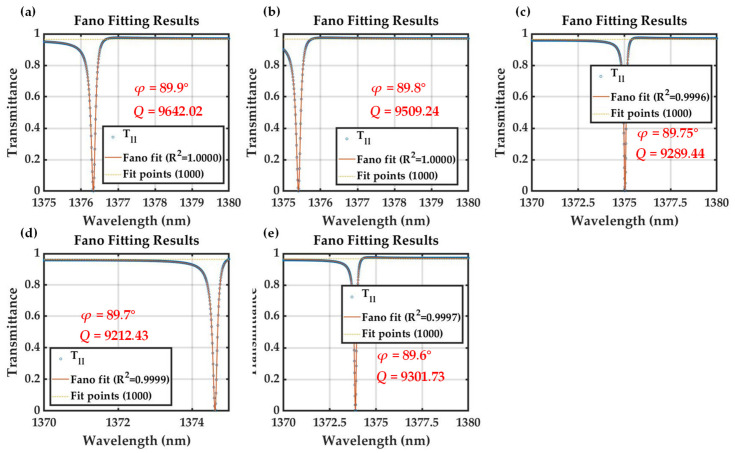
Fano fitting results of the T_ll_ spectra under different sidewall angles for the proposed chiral metasurface. (**a**–**e**) Fano fitting results for *φ* = 89.9°, 89.8°, 89.75°, 89.7°, and 89.6°, respectively, under *d*_1_ = 75 nm, *d*_2_ = 35 nm, and *α* = −0.08 rad. The extracted *Q* factors are 9642.02, 9509.24, 9289.44, 9212.43, and 9301.73, respectively.

Subsequently, we analyzed the influence of sidewall-angle variation on the CD and *Q* factor of the proposed chiral metasurface. The structure with *d*_1_ = 75 nm, *d*_2_ = 35 nm, and *α* = −0.08 rad was again taken as an example. The sidewall angle is denoted as φ, as shown in Figure 11a. We calculated the CD and *Q* factor for *φ* = 89.9°, 89.8°, 89.75°, 89.7°, and 89.6°. As shown in Figure 11b–f and Figure 12a–e, the corresponding CD values are −0.9840, −0.9791, −0.9755, −0.9673, and −0.9590, respectively; the corresponding *Q* factors are 9642.02, 9509.24, 9289.44, 9212.43, and 9301.73, respectively.

**Figure 13:**
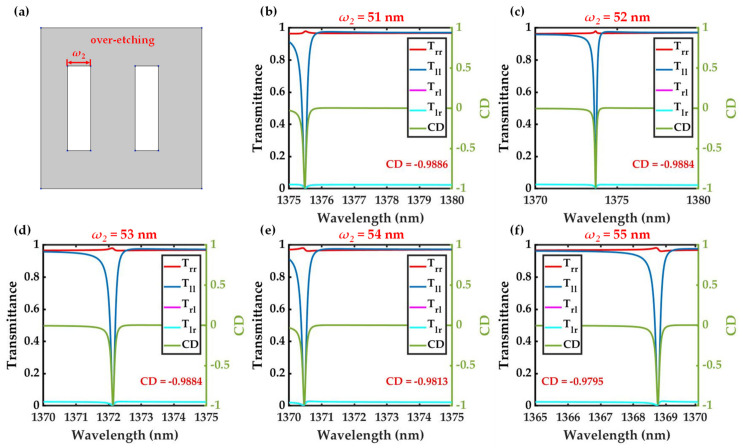
Influence of over-etching on the chiral response of the proposed metasurface. (**a**) Schematic of the air-groove linewidth *ω*_2_ after over-etching. (**b**–**f**) Transmission spectra and CD responses for *ω*_2_ = 51, 52, 53, 54 and 55 nm, respectively, under *d*_1_ = 75 nm, *d*_2_ = 35 nm, and *α* = −0.08 rad.

**Figure 14:**
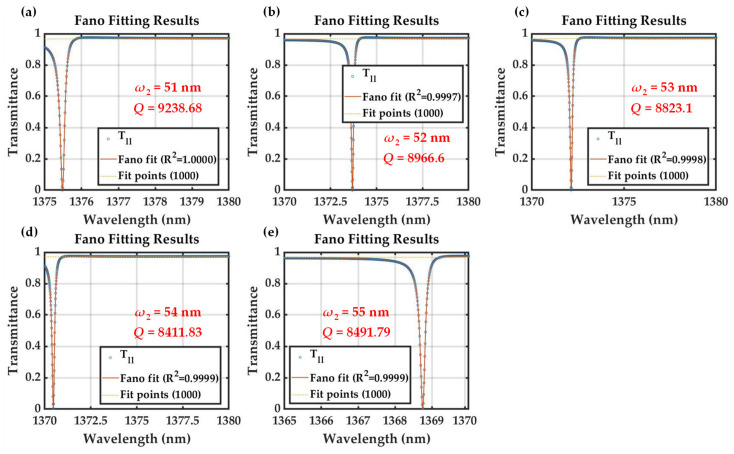
Fano fitting results of the T_ll_ spectra under different over-etching conditions for the proposed chiral metasurface. (**a**–**e**) Fano fitting results for *ω*_2_ = 51, 52, 53, 54, 55 nm, respectively, under *d*_1_ = 75 nm, *d*_2_ = 35 nm, and *α* = −0.08 rad. The extracted *Q* factors are 9238.68, 8966.60, 8823.10, 8411.83, and 8491.79, respectively.

Finally, we investigated the effect of over-etching on the CD and *Q* factor. For the same chiral metasurface, namely *d*_1_ = 75 nm, *d*_2_ = 35 nm, and *α* = −0.08 rad, the linewidth of the air groove after over-etching is denoted as *ω*_2_, as shown in Figure 13a. We calculated the CD and *Q* factor for *ω*_2_ = 51, 52, 53, 54, and 55 nm. As shown in Figure 13b–f and Figure 14a–e, the corresponding CD values are −0.9886, −0.9884, −0.9884, −0.9813, and −0.9795, respectively; the corresponding *Q* factors are 9238.68, 8966.60, 8823.10, 8411.83, and 8491.79, respectively.

In practical experimental implementations, the metasurface is usually supported by a substrate rather than suspended in a symmetric refractive-index environment. The introduction of a substrate breaks the out-of-plane refractive-index symmetry and may therefore modify the quasi-BIC resonance and the corresponding chiral response. To evaluate the influence of a realistic supporting layer, a SiO_2_ substrate was introduced into the simulation model. The refractive index of the SiO_2_ substrate was set to 1.46, with a thickness of 2250 nm and both the length and width set to 750 nm.

**Figure 15:**
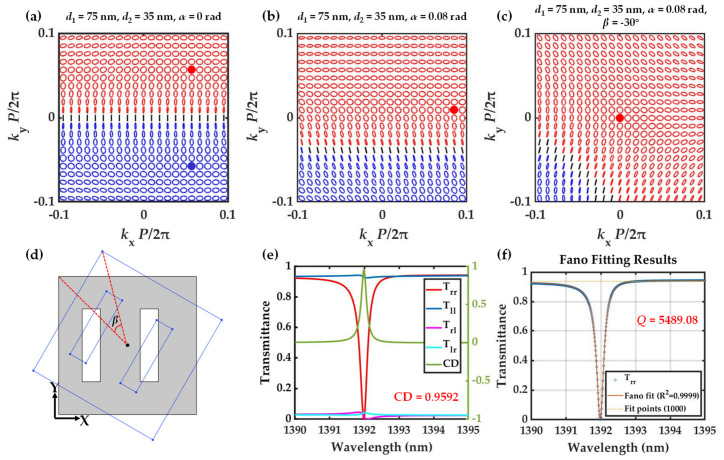
Influence of the SiO_2_ substrate on the chiral quasi-BIC response of the proposed metasurface. (**a**) C-point distribution of the substrate-supported structure with *d*_1_ = 75 nm, *d*_2_ = 35 nm, and *α* = 0 rad. (**b**) C-point distribution with *d*_1_ = 75 nm, *d*_2_ = 35 nm, and *α* = 0.08 rad. (**c**) C-point distribution after introducing the rotation angle *β* = −30°, where the C_+_ point is shifted to the Г point. (**d**) Schematic of the rotation angle *β* around the *z*-axis. (**e**) Transmission spectra and CD response of the optimized substrate-supported structure. (**f**) Fano fitting result of the T_rr_ spectrum, with an extracted *Q* factor of 5489.08. In (**a**–**c**), the red and blue ellipses and circular dots indicate opposite elliptical polarization states and the corresponding C points, respectively, while the black lines indicate linear polarization. In (**d**), the blue boxes show the rotated unit cell, the red dashed lines indicate the rotation angle *β*.

In the substrate-supported structure, when *d*_1_ = 75 nm, *d*_2_ = 35 nm, and *α* = 0 rad, the two C points are located at *k*_x_*P*/2π = 0.07, *k*_y_*P*/2π = 0.06 and *k*_x_*P*/2π = 0.07, *k*_y_*P*/2π = −0.06, respectively, as shown in Figure 15a. Compared with the suspended structure, the introduction of the SiO_2_ substrate breaks the refractive-index symmetry along the z direction, causing the distribution of the two C points to deviate from the *k*_x_*P*/2π = 0 axis.

Furthermore, when *d*_1_ = 75 nm, *d*_2_ = 35 nm, and *α* = 0.08 rad, the C_+_ point is located at *k*_x_*P*/2π = 0.09 and *k*_y_*P*/2π = 0.01, as shown in Figure 15b. To tune the C_+_ point to the Г point, we introduce a third tuning parameter, *β*, defined as the rotation angle around the *z*-axis, as shown in Figure 15d. When *d*_1_ = 75 nm, *d*_2_ = 35 nm, *α* = 0.08 rad, and *β* = −30°, the C_+_ point can be shifted to the Г point, as shown in Figure 15c.

Therefore, after introducing the SiO_2_ substrate, a strong chiral response can still be recovered by further tuning the structural rotation angle *β*. As shown in Figure 15e, the maximum CD reaches 0.9592. In addition, we performed Fano fitting on the simulated T_rr_ spectrum, and the fitting result is shown in Figure 15f. The fitted *Q* factor is 5489.08. These results indicate that, although the SiO_2_ substrate breaks the out-of-plane symmetry and modifies the distribution of the C points, the substrate-supported metasurface can still achieve a chiral quasi-BIC response with high CD and a relatively high *Q* factor through further optimization of the structural parameters.

To further clarify the novelty and advantages of the proposed groove-based geometry, Table 1 compares the proposed metasurface with representative chiral BIC metasurfaces reported in recent literature.

As shown in Table 1, the proposed structure achieves near-unity CD values of −0.98/0.98 while maintaining high *Q* factors of 9446/9635. The corresponding operating bandwidths are 23/22.6 GHz. In addition, by integrating graphene, the proposed structure enables active tuning in the Fermi-level range of *E*_F_ = 0.35–0.55 eV, whereas most reported chiral BIC metasurfaces are passive. Therefore, the proposed groove-based geometry offers a competitive combination of high CD, high *Q* factor, relatively simple structural design, and active tunability.

Overall, these results indicate that strong chiral response can be achieved by combining the polarization singularity of a symmetry-protected BIC with controlled in-plane and out-of-plane symmetry breaking. Unlike conventional chiral metasurfaces that usually rely on intrinsically three-dimensional chiral geometries or strong polarization conversion, the present structure achieves |CD| close to 1 mainly through spin-selective co-polarized coupling to a quasi-BIC mode. The evolution of the C points in momentum space provides a clear physical picture for understanding the origin of the chiral response and also offers a useful route for designing angle-dependent polarization selectivity. In addition, the introduction of graphene converts the transmission-type chiral response into a tunable absorption response. This feature may be valuable for reconfigurable chiral filters, polarization-selective absorbers, and polarization-sensitive photodetectors. Future work may further focus on the experimental fabrication of graphene–metasurface hybrid systems and the extension of this mechanism to multiband chiral devices.

## 4. Conclusions

In this work, a symmetry-broken silicon metasurface was proposed to realize strong chiral responses based on quasi-BIC modes. The fully symmetric structure supports a symmetry-protected BIC at the Г point with a polarization vortex in momentum space. After breaking the in-plane C_2_ symmetry, the polarization singularity splits into two C points with opposite handedness. By further introducing out-of-plane asymmetry, one of the C points can be shifted to the Г point, enabling spin-selective coupling between circularly polarized light and the quasi-BIC mode. Reversing the out-of-plane tilt direction switches the handedness of the chiral response, with |CD| approaching 1.

The transmission analysis shows that the strong circular dichroism mainly originates from selective suppression of the co-polarized transmission channel rather than cross-polarization conversion. Multipole decomposition confirms that the chiral quasi-BIC resonance is dominated by the magnetic dipole response. In addition, the proposed structure exhibits angle-dependent circular dichroism under oblique incidence, and the integration of monolayer graphene further converts the transmission-type chiral response into a tunable chiral absorption response. This work provides a possible route for designing tunable chiral metasurfaces, polarization-selective absorbers, and polarization-sensitive optoelectronic devices.

Although the proposed graphene–silicon chiral quasi-BIC metasurface exhibits strong spin-selective chiral response and tunable chiral absorption in numerical simulations, several practical issues should be further considered. Specifically, the fabrication-tolerance analysis shows that, under small geometrical perturbations, including groove rounding, sidewall-angle variation, and over-etching, the proposed chiral quasi-BIC metasurface can still maintain relatively high CD and *Q* factors. This indicates that the chiral response is not destroyed by slight geometrical deviations and that the structure exhibits a certain degree of fabrication robustness. In addition, the simulation results with a SiO_2_ substrate show that, in the presence of a practical supporting layer, the structure can still maintain a strong CD response by introducing an in-plane rotation angle β around the *z*-axis. However, because the substrate breaks the ideal out-of-plane refractive-index symmetry, the *Q* factor decreases. Therefore, although the spin-selective chiral response of the proposed structure exhibits certain robustness, maintaining a high-*Q* quasi-BIC resonance in practical substrate-supported structures remains an important issue to be further addressed.

For the graphene-integrated structure, the practical implementation of electrical gating also requires further consideration. Although tuning the Fermi level of graphene can effectively modulate the dissipative loss and realize tunable chiral absorption, practical gate control requires the introduction of gate electrodes, gate dielectric layers, and electrical contact structures [47]. These additional components may perturb the localized optical field of the chiral quasi-BIC mode, or introduce parasitic absorption and scattering losses, thereby affecting the *Q* factor, chiral response, and absorption modulation depth. Therefore, future work should focus on the co-design of the optical metasurface, supporting substrate, and electrical gating configuration, including the use of low-loss substrates or membranes, low-loss transparent electrodes, optimized gate dielectric layers, and the placement of contact electrodes away from regions with strong localized fields. These efforts are essential for advancing the proposed graphene–silicon chiral metasurface from ideal numerical simulations toward experimentally realizable reconfigurable chiral photonic devices.

## Figures and Tables

**Table 1:** Performance comparison between the proposed metasurface and recently reported chiral BIC metasurfaces.

Ref.	Materials	Peak CD	*Q* Factor	Operating Bandwidth	Active Tuning Range
[42]	Si	−0.87/0.91	3873/7196	13/7.2 GHz	No
[43]	Si/graphene	−0.954/0.939	2917/1433	0.153/0.32 GHz	*E*_F_ = 0.05–1 eV
[44]	LiTaO_3_	−0.98/0.98	862/2673	2.8/0.9 GHz	No
[45]	TiO_2_	0.93	2663	180 GHz	No
[46]	Si	0.7	10^4^	0.06 GHz	No
This work	Si/graphene	−0.98/0.98	9446/9635	23/22.6 GHz	*E*_F_ = 0.35–0.55 eV

## Data Availability

Data are contained within the article.
